# Reaping the Fruits of LLM Pruning: Towards Small Language Models for Efficient Non-Coding Variant Effect Prediction

**DOI:** 10.3390/genes16111358

**Published:** 2025-11-10

**Authors:** Megha Hegde, Jean-Christophe Nebel, Farzana Rahman

**Affiliations:** 1 School of Computer Science and Mathematics, Kingston University, London KT1 2EE, UK; megha.hegde@kingston.ac.uk (M.H.); farzana@kingston.ac.uk (F.R.); 2 Holmwood House, Grove Crescent, Kingston Upon Thames KT1 2EE, UK

**Keywords:** variant effect prediction, large language model, small language model, non-coding DNA, deep learning

## Abstract

**Background**: Interpreting variant effects is essential for precision medicine. Large Transformer-based genomic language models (DNABERT 2, Nucleotide Transformer) capture patterns in coding DNA but scale poorly for non coding variant prediction because attention complexity grows quadratically with sequence length. Evidence from natural language processing shows that pruning less informative layers can reduce model size and computational load without sacrificing accuracy. **Methods**: We systematically ablated each Transformer layer in DNABERT 2 and the Nucleotide Transformer to assess its contribution to variant prediction. By observing changes in performance, we built layer importance profiles and created pruned models by removing redundant layers. Pruned and full models were fine tuned with identical hyperparameters using the Enformer eQTL causal variant dataset, a curated benchmark for non coding variant effect prediction. **Results**: Layer ablation revealed that the importance of individual layers varies widely across models; some layers can be removed with little loss in performance while others are critical. After fine tuning, pruned models achieved accuracy and area under the ROC curve comparable to full models. Additionally, pruned versions required substantially less training time and memory, reducing resource usage by a significant margin. **Conclusions**: Layer wise pruning provides a principled strategy for developing compact genomic LLMs. By identifying and removing less critical layers, we produced leaner models that preserve predictive power while lowering computational demands. These efficient models demonstrate how insights from general LLM research can advance genomic variant interpretation and make large scale non coding analysis more accessible in research and clinical settings. This approach complements ongoing efforts to optimise Transformer architectures for genomic data.

## 1. Introduction

In the era of personalised healthcare, interpreting the effects of variants in human DNA has become increasingly important to inform treatment and predict outcomes [1,2]. Machine learning and deep learning models in particular have become widely used to support this endeavour, as they are well-suited to analyse the enormous amounts of genomic data produced by next-generation sequencing technologies [3,4,5].

Due to similarities in the structure of natural languages and genomic/proteomic sequences, natural language processing techniques have demonstrated significant applicability in bioinformatics [6,7]. Since the advent of the Transformer, large pretrained language models, called biological foundation models, have become widespread for solving a variety of bioinformatics tasks. Among them, large language models (LLMs) have been widely used for variant effect prediction, achieving promising results in many cases. However, recent reviews of the field have identified some key challenges that have yet to be addressed [8,9]. While LLMs have achieved good results on variants within the coding regions of the human genome, non-coding variants are still underexplored, and results on such problems are sub-optimal [10,11]. Furthermore, the problem of non-coding variant effect prediction necessitates longer-range context, and hence, it requires longer DNA sequences as input to the model. However, due to the attention mechanism’s quadratic scaling of computational complexity with sequence length, Transformer-based LLMs are highly inefficient for longer-context (1000+ bp) problems. Recent research has explored alternatives to the attention mechanism, such as Hyena and Mamba; however, models using these alternatives still produce unsatisfactory results on non-coding variant effect prediction [11,12,13]. Hence, Transformer-based LLMs remain the prevalent technology in the field.

As suggested by their name, LLMs are defined by their immense scale, typically comprising anywhere from a dozen to several hundred layers and containing millions to billions of parameters. This is equally true for genomic LLMs; for instance, the widely used Nucleotide Transformer series [14] contains models with up to 2.5 billion parameters. Although genomic LLMs have been steadily increasing in size in the pursuit of better modelling biological information, recent research has shown that the relationship between the number of layers and the context captured by a model is not as straightforward as previously hypothesised [15,16]. In particular, a number of papers have reported that not all layers are equal, with different layers learning different amounts and types of information [17,18]. Crucially, multiple studies have evidenced that pruning (removing) some layers of a model drastically impacts the model’s performance on downstream tasks, whereas the pruning of others has a negligible effect [18,19,20]. The most important layers have been referred to “cornerstone” layers [18], whose removal causes a “significant performance drop”.

While these studies have made significant strides in uncovering the inner workings of LLMs, they have focused on English language models. In contrast, such research is still lacking for genomic LLMs. As personalised medicine becomes more mainstream, it is crucial for the research community to work together to make models which are accurate, efficient, and unbiased. However, existing genomic LLMs often take days or weeks to train, using multiple GPUs, hence making them inaccessible to researchers without high-performance computing infrastructure. Fully understanding the composition of LLMs and deciphering the impact of each layer will enable the creation of streamlined models that are more computationally efficient, broadly accessible, and suitable for deployment in clinical environments.

This study investigates the impacts of different LLM layers on downstream performance by performing layer-wise ablation of two modern genomic LLMs, DNABERT-2 [21] and the Nucleotide Transformer [14]. Consistent with findings in natural language models, the results demonstrate that layer pruning can reduce fine-tuning time while maintaining or even improving model performance.

## 2. Materials and Methods

This study aims to support the production of efficient language models for the prediction of human non-coding DNA variants. The role and/or redundancy of different LLM layers in modelling DNA sequences is investigated by performing systematic layer-wise pruning of DNABERT-2 [21] and Nucleotide Transformer [14], which have both been widely used for DNA variant effect prediction tasks. By doing so, the study investigates whether structured pruning can preserve LLM performance while reducing fine-tuning and evaluation time, as has been demonstrated for natural language processing tasks [18,19,20]. The models are trained and evaluated on the relevant splits of the eQTL variant data derived from the Enformer paper [3]. The task explored is the binary classification of single-nucleotide variants as pathogenic or benign from a single DNA sequence input. The dataset details are summarised in Table 1.

**Figure 1:**
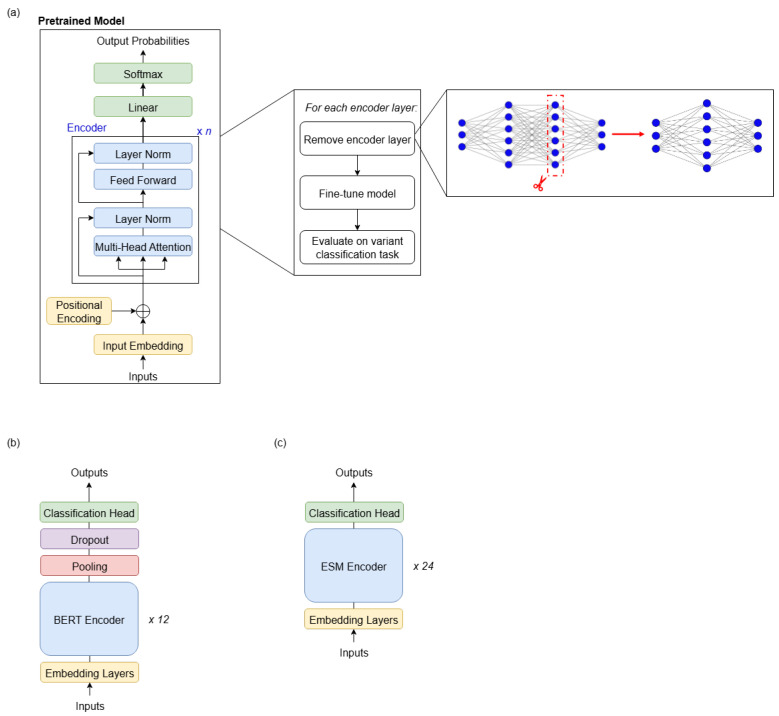
(**a**) Methodology used in this study. For each layer in the encoder, a copy of the pretrained model was created with the desired encoder layer removed. This copy of the model was then fine-tuned and evaluated on the two non-coding variant classification benchmark datasets. (**b**) Architecture of DNABERT-2 as described in [21]. This is an encoder-only Transformer model, with 12 encoder layers. (**c**) Architecture of Nucleotide Transformer as described in [14]. This is an encoder-only Transformer model, with 24 encoder layers.

The methodology shown in Figure 1a was followed for both state-of-the-art DNA LLMs. In each experiment, a copy of the pretrained model is created and the desired layer removed. This copy is then fine-tuned on the 88,717 variant-centred DNA sequences in the training split of the eQTL dataset [3]. The initial goal was to repeat each experiment for each model three times, with different random seeds, on the same hardware. However, due to the time-consuming nature of the experiments, it was necessary to train the models on two different machines. Each DNABERT-2 model was trained until convergence of the validation loss and repeated three times with different random seeds. The training was performed on 2 × Quadro RTX 8000 GPUs, taking around 1 h per pruned model on average. The Nucleotide Transformer had a much larger size and hence a significantly longer training time; training to convergence on 2 × NVIDIA TITAN Xp GPUs took approximately 19 h. Hence, due to time and resource constraints, it was not possible to train each version of the model to convergence three times with different random seeds. Instead, to make experiments manageable, each model was trained for 5 epochs, which was the minimal number of epochs to achieve an AUROC above 70%, i.e., substantially better than random, on the eQTL variant classification task (Table 1). This reduced the fine-tuning time for each model to approximately 7 h. Repeated runs were performed for the original (un-pruned), cornerstone, and best models with three different random seeds, demonstrating good agreement (Table A1, Table A2 and Table A3).

Enformer [3] itself maintains the state-of-the-art performance on the eQTL dataset. However, technical constraints prevented the use of this model in the study. Additionally, it is important to note that the Enformer methodology differs from that described above. Rather than fine-tuning the pretrained LLM, the embeddings are instead extracted and used as feature inputs for a random forest classifier. The figures quoted for this model are derived from the literature.

Finally, the models were evaluated on the binary variant classification task, across the 8846 variant-centred DNA sequences in the evaluation split of the eQTL dataset. Instructions for accessing the training and evaluation data are included in the *Data Availability* section of this paper. It should be noted that the training and evaluation datasets are both balanced, containing an equal number of samples in the positive and negative classes. The model architectures for DNABERT-2 and Nucleotide Transformer are summarised in Figure 1b and Figure 1c, respectively, and key details are highlighted in Table 2. DNABERT-2 was pretrained on human and multispecies reference genomes. The variant of Nucleotide Transformer used in this study was pretrained on the human reference genome only. Instructions for accessing the pretrained models are available in Appendix A.

As is standard in the field, the models were assessed across multiple metrics during evaluation, i.e., accuracy, AUROC, and F1 score. The rates of true and false positives and negatives were also recorded for each experiment.

For each model, “cornerstone” and “unfavourable” layers were identified via comparison to the baseline model (i.e., the original model with no layers removed). As per the definition in [18], **cornerstone** layers are those that significantly contribute to the model’s performance across all metrics. Ref. [18] quantifies this by selecting these as layers which, when removed, result in the model’s performance dropping to random. However, as no such layers exist in the models used here, *we instead quantify cornerstone layers as those which result in all metrics being at least 5% worse than the baseline when removed*. **Unfavourable** layers were identified as those that either had a negligible impact on the downstream prediction or in fact made it worse. When individually removed, these layers resulted in all metrics being better than or equal to the baseline. A reduction of within one standard deviation of the average metric observed across the three runs was considered to be of equal performance to the baseline. Hence, the criteria used were that at least three out of the four equations in (1) must be fulfilled. Here, $\mu_{metric}$ refers to the mean of a metric recorded across three runs, _b refers to the baseline (un-pruned) model, and _layer refers to the model with a specific layer pruned. The percentage of true positives, %TP, is calculated as the number of true positives over the total number of positive samples.

(1a) $$\begin{array}{c} \mu_{acc\_b}\leq \mu_{acc\_layer} \end{array}$$

(1b) $$\begin{array}{c} \mu_{auroc\_b}\leq \mu_{auroc\_layer} \end{array}$$

(1c) $$\begin{array}{c} \mu_{f1\_b}\leq \mu_{f1\_layer} \end{array}$$

(1d) $$\begin{array}{c} \mu_{\%TP\_b}\leq \mu_{\%TP\_layer} \end{array}$$

Versions of the models (a) removing all the unfavourable layers and (b) removing all non-cornerstone layers were fine-tuned and evaluated on the downstream task, and compared to the baseline.

## 3. Results

**Figure 2:**
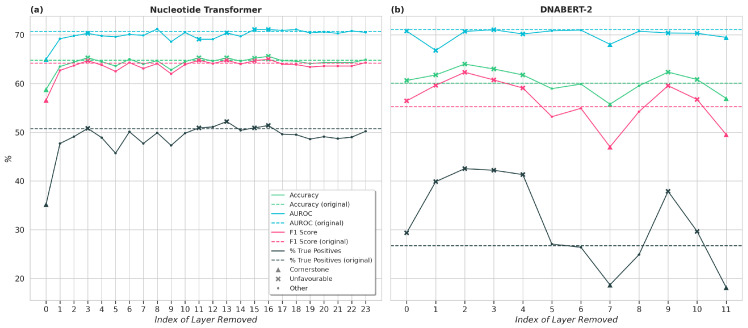
Results of layer-wise pruning of (**a**) Nucleotide Transformer and (**b**) DNABERT-2 on the results of the Enformer eQTL variant classification task. Reported values for DNABERT-2 are averaged across three runs with different random seeds. The percentage of true positives is defined as %TP=TP/(TP+FN)∗100.

Figure 2 provides an overview of the layer-wise pruning results for both models, with Figure 2a illustrating outcomes for DNABERT-2 and Figure 2b for the Nucleotide Transformer. Both models exhibited marked variation across layers, enabling the identification of cornerstone layers with significant impacts on performance and unfavourable layers with minimal or negative impact on performance. Despite observable trends within each model, no consistent pattern emerges across DNABERT-2 and the Nucleotide Transformer. While both sets of results suggest that the final layers may be less influential, the impact of pruning early and intermediate layers varies considerably between the models. This lack of consistency underscores the need for a deliberate and informed pruning strategy, as random layer removal is unlikely to yield performance improvements. A common feature across the results of both models is accuracy and F1 scores that are consistently lower than the AUROC. Investigation of the results shows that, with both architectures used, the rate of true negatives is higher than the rate of true positives, and the rate of false negatives is comparatively higher than the rate of false positives. Hence, the models have a greater tendency to predict negative (benign) labels compared to positive (pathogenic) labels. The slight imbalance in the dataset used (Table 1) may contribute to this. Future experiments with better thresholding or including further metrics may resolve this issue [22,23,24].

### 3.1. DNABERT-2

The experiments for DNABERT-2 demonstrate a significant difference between the layers of the encoder. Layers 7 and 11 were identified as “cornerstone” layers, leading to significantly poorer performance when individually removed (Figure 2). A version of the model preserving only the cornerstone layers achieved slightly improved performance on the downstream task compared to the original (un-pruned) model, while decreasing inference time by 32%. However, fine-tuning time in fact *increased* by 33% for this model, as the validation loss took longer to converge. This reflects previous studies in the field which indicate that smaller models may have less stable convergence patterns and may hence take more gradient updates to converge [16,25,26].

Layers 0, 1, 2, 3, 4, 9, and 10 were identified as “unfavourable” layers. A version of the model with all unfavourable layers removed, when fine-tuned, performed similarly on the evaluation task compared to the original (un-pruned) model, while significantly reducing fine-tuning (−51%) and evaluation (−33%) times (Table 3). A summary of the numbers of different types of layers identified is provided in Table 4.

### 3.2. Nucleotide Transformer

The experiments for the Nucleotide Transformer once again showed a marked difference between the various encoder layers. Layers 3, 11, 13, 15, and 16 were identified as “unfavourable” layers, resulting in unchanged or better performance across all metrics when individually removed. As shown in Table 5, a version of the model with both unfavourable layers removed resulted in slightly better performance on the evaluation task compared to the baseline (un-pruned) model. The experiments identified the cornerstone layer as 0. A model preserving only this cornerstone layer was able to achieve results within 5% of that of the original model, while drastically reducing both the fine-tuning and evaluation times by 95% (Table 5). The reduced fine-tuning time is in contrast to what was observed for DNABERT-2; this is likely because the Nucleotide Transformer was fine-tuned for a set number of epochs rather than to convergence. The results indicate that, while this layer holds high importance, other layers also make a valuable contribution to an accurate downstream prediction. The numbers of each type of layer identified in the model are provided in Table 6.

### 3.3. Comparison with State-of-the-Art

Comparison with Enformer shows that a pruned version of DNABERT-2 is able to produce results close to the state of the art while using less than 50% of the number of parameters (Table 7). This reinforces the idea that informed pruning strategies can lead to good performance with lower computational cost.

## 4. Discussion and Conclusions

As demonstrated in [18], the layer-wise pruning experiments evidenced the existence of “cornerstone” layers whose removal significantly degraded the models’ performance. Preserving only the cornerstone layers, both models were able to maintain results within 5% of the original model. For Nucleotide Transformer, this approach significantly reduced the fine-tuning and evaluation time. For DNABERT-2, however, the fine-tuning time in fact increased, as the model took longer to converge. This suggests that aggressive pruning does not correlate exactly with model efficiency, and reflects findings from previous studies that very small models may face issues with convergence [26]. Additionally, the decrease in performance on the evaluation task resulting from this aggressive pruning was not insignificant, suggesting that non-cornerstone layers still play a key role in modelling context required for the downstream task.

For both models investigated, multiple layers were identified which, when removed, resulted in similar or better performance on the evaluation task; these were termed “unfavourable“ layers. When versions of the models with all unfavourable layers were fine-tuned, their performance on the evaluation task in fact improved, while the training and evaluation times were significantly reduced. This improvement in results with layer removal suggests the existence of redundancy within the removed layers. Such behaviour has previously been observed in similar experiments on LLMs for natural language processing [27]. In both cases, removing all unfavourable layers from the model resulted in similar or better performance on the downstream task, while reducing fine-tuning and evaluation times.

Though the existence of cornerstone and unfavourable layers was clear across both models, there was no consistent pattern observed regarding which parts of the model were most relevant to the downstream task. This suggests that layer importance varies significantly by model architecture. While the layer-wise ablation here was able to identify the most and least important layers, it is a time-consuming task, and hence, it is a sub-optimal method for estimating layer-wise importance for enhancing model efficiency. However, this work provides a basis for comparison with layer-wise importance estimation methods established in the natural language field [28,29] to test whether they are applicable to genomic language models.

Comparison with the state of the art [3] demonstrated that the pruned models produced in this study achieved close to state-of-the-art performance, and DNABERT-2 did so while using fewer than 50% of the number of parameters. This finding aligns with recent large language model studies indicating that larger model size does not necessarily translate to superior downstream task performance [30,31]. While technical constraints prevented the use of the state-of-the-art model for this problem, the results suggest that distinct cornerstone and unfavourable layers are likely to exist across genomic language models with multiple encoder layers. Future research will investigate the application of structured pruning techniques to state-of-the-art models to determine whether targeted layer optimisation can yield further performance improvement.

This work provides a basis for further exploration of how LLM efficiency can be enhanced in order to enable large-scale genomic studies. However, it is crucial to consider how efficiency and accuracy should be balanced, particularly in clinical settings, where incorrect predictions can be devastating.

## Figures and Tables

**Table 1:** Key details of the datasets used in this study.

Dataset	Used For	No. of Benign Samples	No. of Pathogenic Samples
Enformer eQTL data (train split)	Fine-tuning	44,655 (50.1%)	44,405 (49.9%)
Enformer eQTL data (test split)	Evaluation	4306 (48.6%)	4556 (51.4%)

**Table 2:** Key details of the two models used in these experiments.

Model	No. of Params (Pretrained Model)	No. of Params (After Fine-Tuning)	No. of Encoder Layers
DNABERT-2	117 million	119 million	12
Nucleotide Transformer	480 million	482 million	24

**Table 3:** Results of layer-wise pruning of the DNABERT-2 [21] model. Values given are for binary classification of non-coding variants from an eQTL dataset [3]. * Removing all “unfavourable” layers, i.e., those which have positive or no effect on the evaluation metrics when removed. ^+^ Preserving only the “cornerstone” layers, i.e., those which lead to a significant drop in performance when removed. Values provided are averaged over three runs using different random seeds. Full results are provided in the Supplementary Materials.

Layer Index Removed	Fine-Tuning Time (Proportion of Original)	Evaluation Time (Proportion of Original)	Accuracy (%)	AUROC (%)	F1 (%)
None	1	1	60.07	71.07	55.27
0, 1, 2, 3, 4, 9, 10 *	0.49	0.77	61.16	70.43	57.38
0–6 & 8–10 ^+^	1.33	0.68	62.16	67.32	60.85

**Table 4:** Numbers of different types of layers identified in DNABERT-2.

Layer Type	Number of Layers
All Transformer layers	12
Cornerstone	2
Unfavourable	7
Other	3

**Table 5:** Results of layer-wise pruning of the Nucleotide Transformer [14] model. Values given are for binary classification of non-coding variants from an eQTL dataset [3]. * Removing all “unfavourable” layers, i.e., those which have positive or no effect on the evaluation metrics when removed. ^+^ Preserving only the “cornerstone” layers, i.e., those which lead to a significant drop in performance when removed. Full results are displayed in the Supplementary Materials.

Layer Index Removed	Fine-Tuning Time (Proportion of Original)	Evaluation Time (Proportion of Original)	Accuracy (%)	AUROC (%)	F1 (%)
None	1	1	64.76	70.70	64.19
3, 11, 13, 15, 16 *	0.80	0.79	65.59	71.33	65.19
1–23 ^+^	0.05	0.05	62.79	67.23	62.34

**Table 6:** Numbers of different types of layers identified in the Nucleotide Transformer.

Layer Type	Number of Layers
All Transformer layers	24
Cornerstone	1
Unfavourable	5
Other	18

**Table 7:** Comparison of the best results on the eQTL dataset [3] produced in this study with the state of the art. N.B.: the Enformer model uses a different methodology to this study, and the figures quoted are taken from the literature [3].

Model Description	No. of Parameters	No. of Encoder Layers	AUROC (%)
Enformer as per [3]	249 million	11	74.7
DNABERT-2 with unfavourable layers removed	119 million	8	71.5
Nucleotide Transformer with unfavourable layers removed	482 million	22	72.1

**Figure A1:**
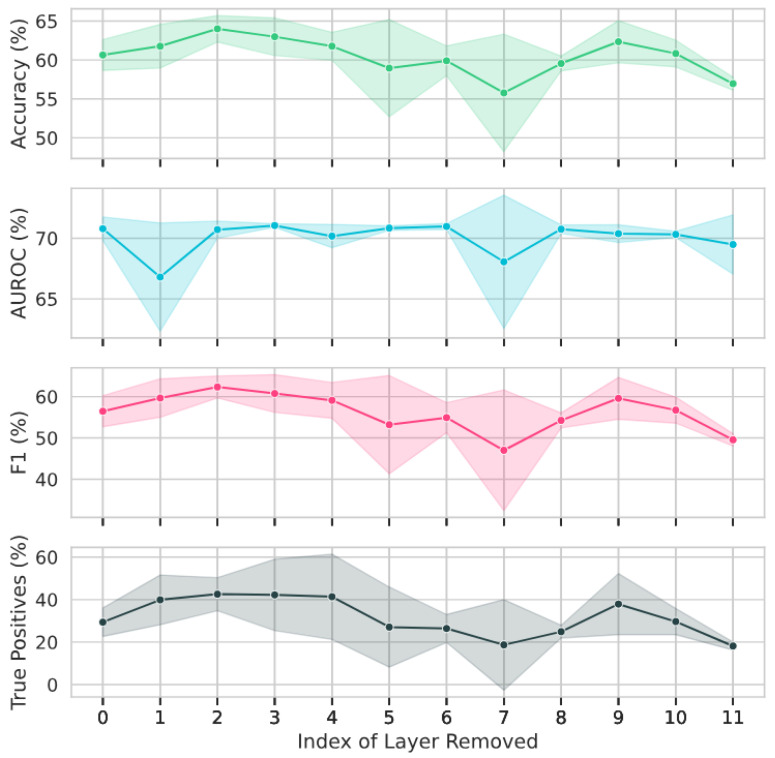
Results of layer-wise pruning of DNABERT-2 on the results of the Enformer eQTL variant classification task. The line represents the average across three runs with different random seeds, and the error bars represent the standard deviation. The percentage of true positives is defined as %TP=TP/(TP+FN)∗100.

## Data Availability

The training and evaluation datasets are derived from the eQTL causal variant dataset from [3]. The version of the dataset used in this paper is available on Huggingface as part of the “Genomics Long-Range Benchmark” and can be found at the following link: https://huggingface.co/datasets/InstaDeepAI/genomics-long-range-benchmark (accessed on 29 September 2025).

## Supplementary Materials

Supplementary files containing the full results are available on Zenodo at the following link: https://doi.org/10.5281/zenodo.17434133. The code is available on GitHub at the following link: https://github.com/meghegde/dna_llm_pruning.

## Appendix A

### Appendix A.1. Details of Pretrained Models Used

The pretrained models were loaded from the official Huggingface (https://huggingface.co/, accessed on: 29 September 2025) repositories. DNABERT-2 is an encoder-only Transformer-based model, pretrained on multispecies reference genome data. The version used can be found on Huggingface under the reference “zhihan1996/DNABERT-2-117M”. The Nucleotide Transformer paper produced a series of encoder-only Transformer-based DNA LLMs, pretrained on different genomes. The version used in these experiments can be found on Huggingface under the reference “InstaDeepAI/ nucleotide-transformer-500m-human-ref” and was pretrained only on the human reference genome.

### Appendix A.2. Details of Repeated Runs for DNABERT-2

The hyperparameters used for DNABERT-2 are as listed below. For any hyperparameters not listed, the default values were used.

Parameter-efficient fine-tuning using the peft (https://pypi.org/project/peft/, accessed on: 29 September 2025):

- r=1;
- lora_alpha=32;
- lora_dropout=0.1.

Huggingface TrainingArguments (https://huggingface.co/docs/transformers/en/main_classes/trainer#transformers.TrainingArguments, accessed on: 29 September 2025):

- per_device_train_batch_size=8;
- per_device_eval_batch_size=8;
- gradient_accumulation_steps=32;
- warmup_steps=500;
- logging_strategy=′*epoch*′;
- eval_strategy=′*epoch*′;
- eval_steps=100;
- save_steps=100.

### Appendix A.3. Details of Repeated Runs for Nucleotide Transformer

The hyperparameters used for Nucleotide Transformer are as listed below. For any hyperparameters not listed, the default values were used.

Parameter-efficient fine-tuning using the peft (https://pypi.org/project/peft/, accessed on: 29 September 2025) package:

- r=1;
- lora_alpha=32;
- lora_dropout=0.1.

**Table A1:** Results of repeated runs fine-tuning the original (non-pruned) Nucleotide Transformer [14] model for 5 epochs. As shown, the results from each run were the same up to two decimal places. Results given are for binary classification of non-coding variants from the Enformer eQTL dataset [3].

Seed	Training Time (Hours)	Evaluation Time (Minutes)	Accuracy (%)	AUROC (%)	F1 (%)
41	7.53	8.49	64.76	70.70	64.19
42	7.50	8.51	64.76	70.70	64.19
43	7.48	8.49	64.76	70.70	64.19

**Table A2:** Results of repeated runs fine-tuning the Nucleotide Transformer [14] model for 5 epochs, with all unfavourable layers removed. As shown, the results from each run were the same up to two decimal places. Results given are for binary classification of non-coding variants from the Enformer eQTL dataset [3].

Seed	Training Time (Hours)	Evaluation Time (Minutes)	Accuracy (%)	AUROC (%)	F1 (%)
41	6.02	6.73	65.59	71.33	65.19
42	6.02	6.70	65.59	71.33	65.19
41	6.01	6.70	65.59	71.33	65.19

**Table A3:** Results of repeated runs fine-tuning the Nucleotide Transformer [14] model for 5 epochs, preserving only cornerstone layers. Results given are for binary classification of non-coding variants from the Enformer eQTL dataset [3].

Seed	Training Time (Hours)	Evaluation Time (Minutes)	Accuracy (%)	AUROC (%)	F1 (%)
41	0.35	0.42	62.09	67.32	61.55
42	0.35	0.42	63.15	67.18	62.74
43	0.35	0.42	62.09	67.32	61.55

Huggingface TrainingArguments (https://huggingface.co/docs/transformers/en/main_classes/trainer#transformers.TrainingArguments, accessed on: 29 September 2025):

- per_device_train_batch_size=8;
- per_device_eval_batch_size=8;
- gradient_accumulation_steps=32;
- warmup_steps=500;
- fp16=True.
